# Ginger and diabetic nephropathy

**DOI:** 10.12861/jrip.2013.05

**Published:** 2013-03-01

**Authors:** Mahmoud Rafieian-Kopaei, Hamid Nasri

**Affiliations:** ^1^Medical Plants Research Center, Shahrekord University of Medical Sciences, Shahrekord, Iran; ^2^Department of Nephrology, Division of Nephropathology, Isfahan University of Medical Sciences, Isfahan, Iran

**Keywords:** Ginger, Diabetic nephropathy, Renoprotection

**Implication for health policy/practice/research/medical education:**

The combination of metformin and ginger extract may be more effective for the control of diabetes and may have additive protective efficacy on diabetic nephropathy.

Nephropathy of the diabetes is one of the most important complications of this illness. Recently, much attention has been made toward the possible kidney protective properties of ginger in diabetic patients. To find the ameliorative effect of ginger extract against tubular damage induced by gentamicin, we conducted an experimental study, on 50 male Wistar rats, which were allocated into 5 groups of 10 and treated as group I: vehicle, group II: ginger for 3 days then, gentamicin for 7 days, group III ginger orally for 3 days, then ginger plus gentamicin for 7 days, group IV: gentamicin for 7 days. Group V: gentamicin for 10 days, and finally group VI: gentamicin for 7 days, then ginger orally for 10 days. At the end of the study, kidneys were removed for histological evaluation. In this study, we observed that ginger could prevent degeneration of the renal cells and reduce the severity of tubular damage caused by gentamicin. We concluded that ginger is effective as a prophylaxis agent for renal tubular cells against injurious substances acts like gentamicin (1). In the study conducted by Tzeng *et al*, the ameliorative effects of ginger on renal damage in diabetic rat was investigated. In their study, diabetic rats were treated orally with ginger or metformin for 8 weeks. They found that ginger displayed similar characteristics to those of metformin in reducing hyperglycemia and renal dysfunction in diabetic rats. The pathological examinations revealed amelioration of diabetes-induced glomerular pathological changes following the treatment with ginger. Furthermore, the protein expressions of renal nephrin and podocin in diabetic rats were significantly increased following the treatment with ginger. They suggested that the renoprotective effects of ginger may be similar, with the action of metformin, to the prevention of AMP-activated protein kinase protein phosphorylation (2). Metformin has been widely used in the diabetics, especially type II diabetes. Recently, the possible kidney protective role of metformin was been noticed (3). In the study of Morales *et al*, ameliorative properties of metformin against, gentamicin-induced renal tubular damage were shown (4). To find the potential efficacy of metformin to kidney protection against gentamicin-induced acute kidney injury and also to test, whether delay treatment with metformin in acute kidney injury, exerts similar efficacy on gentamicin-renal injury in rats, we conducted a study on Wistar rats .We found that metformin was able to prevent and ameliorate gentamicin-induced acute renal injury. Hence, it might be have renoprotective efficacy (5). More recently, we also observed the efficacy of co-administration of garlic extract and metformin for prevention of gentamicin–renal damage in 70 male Wistar rats (6). The result of these studies shows that metformin and ginger juice has ameliorative effects against gentamicin nephrotoxicity. The protective effect of metformin on diabetic nephropathy was also recently published by kim *et al*. too (7). The incidence of diabetes mellitus and nephropathy of diabetes have risen rapidly (3,7,8).Therefore, the combination of metformin and ginger extract may be more effective for the control of diabetes and may have additive protective efficacy on diabetic nephropathy . In this regard, to better understand the kidney protective properties of Ginger, especially in combination with metformin, more experimental rat models or clinical studies are suggested.

## Authors’ contributions

HN wrote the paper. MRK edited the final draft.

## Conflict of interests

The author declared no competing interests.

## Ethical considerations

Ethical issues (including plagiarism, data fabrication, double publication) have been completely observed by the author.

## Funding/Support

None.
